# Links between food trade, climate change and food security in developed countries: A case study of Sweden

**DOI:** 10.1007/s13280-021-01623-w

**Published:** 2021-09-24

**Authors:** Blaze Horn, Carla Ferreira, Zahra Kalantari

**Affiliations:** 1 Department of Physical Geography and Bolin Centre for Climate Research, Stockholm University, Stockholm, Sweden; 2 Navarino Environmental Observatory, 24001 Messinia, Greece; 3 KTH Royal Institute of Technology, School of Architecture and the Built Environment (ABE), Sustainable Development, Environmental Science and Engineering, Sustainability Assessment and Management, Stockholm University, Stockholm, Sweden

**Keywords:** Climate vulnerability, Developed countries, Food security, Food trade flows, Sourcing countries

## Abstract

Food security is a global concern affecting even highly developed countries. Ongoing globalisation of food systems, characterised by trading interdependencies, means that agricultural production can be disrupted by climate change, affecting food availability. This study investigated Sweden’s food security by identifying major food import categories and associated trade partners (using the World Integrated Trade System database) and vulnerability to frictions in trade deriving from climate change. Vulnerability was assessed through three indicators: exposure based on diversity of sources, dominance and direct trade from supplying countries; sensitivity, assessed using the Climate Risk Index, and adaptive capacity, assessed using the Fragile State Index. The results revealed that Sweden’s grain imports may be most vulnerable, and animal products least vulnerable, to climate change. Management strategies based on this preliminary assessment can be developed by integrating climate vulnerability deriving from food trading into the ‘Gravity’ model, to improve prediction of trade flows.

**Supplementary Information:**

The online version contains supplementary material available at 10.1007/s13280-021-01623-w.

## Introduction

The United Nations (UN) Agenda 2030 provides a vision for achieving global sustainable development (FAO 2020). SDG 2 focuses on hunger, food security, nutrition and sustainable agriculture, with the food security concept gaining particular momentum amongst researchers, governments and the private sector (FAO 2020; UN 2020). There are concerns about how the target ‘Zero Hunger by 2030’ will be achieved (UN 2020).

Food security requires that ‘all people, at all times, have physical, social and economic access to sufficient, safe and nutritious food that meets their dietary needs and food preferences for an active and healthy life’ (FAO 2002). It has four key dimensions: (1) availability, (2) accessibility, (3) utilisation and (4) stability (FAO 2008, 2009).

Nearly 2% of the world’s population, predominantly in Africa, southern Asia and the Caribbean, are classified as being in crisis, emergency or catastrophe conditions, as a result of food consumption gaps, acute malnutrition or food needs being met through depletion of livelihood assets (FSIN 2020). Historically, food shortages have been a challenge confined primarily to the developing world, so research to date has approached food security from the perspective of developing countries. Food security research in developed countries has concentrated on accessibility (socio-economic conditions) and utilisation (malnutrition and obesity) (Ashby et al. 2016; Fusco et al. 2020; Sachs et al. 2020). However, many developed nations also face significant challenges in achieving SDG2 (Sachs et al. 2020).

Developed countries located in climate regions with limited agricultural diversity and insufficient crop yields have become increasingly reliant on trade (Kummu et al. 2020). Globalisation of the food chain is increasing, with food imports today representing three times their value in 2000 (EC 2019). Population increase, environmental degradation, political and economic struggles and climate change are imposing further pressure on the food system (Cottrell et al. 2019; FAO 2020). Trade reliance has resulted in a disconnect between people and nature, with consumers assuming that all their daily resources are abundantly available (Kummu et al. 2020). Since approximately 80% of the world’s population live in import-dependent countries, the risk of food insecurity due to reliance on trade needs to be explored further, to mitigate the risk of food shocks (Porkka et al. 2013; Kummu et al. 2020).

Recent studies have confirmed that observed climate change has affected crop suitability in many areas of the world, including Europe, resulting in changes in production levels of the main agricultural crops, and this trend is expected to continue (Hoegh-Guldberg et al. 2018).

In Sweden, mean annual precipitation is projected to increase by 0–15% (RCP2.6) or 10–35% (RCP8.5) by 2100 (Jordbruksverket 2017). Peak daily average temperature is expected to increase by 1–4 °C, with the lowest daily average temperature rising above 10 °C (Jordbruksverket 2017). These changes are expected to extend the vegetation period by 10–30 days during 2011–2040 and even more by 2100 (Jordbruksverket 2017). As a result, Sweden’s agriculture sector is predicted to benefit from climate change. However, extreme weather events are expected to become more frequent and pose the most significant challenge to Swedish agriculture (Jordbruksverket 2017; Wiréhn 2018). Increased drought during the growing period and heavy precipitation during the harvesting period could have negative impacts on yield (Wiréhn 2018). Maize production in Northern Europe is projected to experience mean yield decreases of between 1 and 14% (RCP8.5) (Wiréhn 2018; Hristov et al. 2020). A warmer and wetter climate could also increase diseases and pest infestations, reducing crop yields (Wiréhn 2018).

Climate change adds complexity to the intricate global system of food interdependency by increasing the risk of disruptions to the stability of agricultural production. Thus, net importers of food, including highly developed countries, are indirectly exposing themselves to climate change occurring beyond their own borders (Cottrell et al. 2019; Kummu et al. 2020). Therefore, presumed ‘secure’ food systems within developed countries need to re-think the effects of climate change (Tendall et al. 2015). This has led to recognition of the food security–climate change nexus (D’Odorico et al. 2014; Benzie et al. 2019; Kummu et al. 2020). In this regard, the Swedish Climate Change Adaptation Network (SCCAN) stresses the need for rapid adaptation of the agriculture sector (SMHI 2021).

To complement the relatively limited research on food security in developed countries, this study investigated their vulnerability to impacts of climate change on food trading, through impacts on trade partners and the pathways taken to import food. Sweden was used as a case study to assess vulnerability to indirect climate impacts associated with food imports, focussing on the availability dimension through trade. Specific objectives of this study were to (i) provide context for Sweden’s current position as a net food importer, both globally and within the European Union (EU); (ii) identify Sweden’s main trade sources and their import contribution to the 10 most relevant food categories and associated sub-categories; (iii) assess the climate vulnerabilities associated with Sweden’s trading partners; and (iv) discuss the impact of these partnerships on Sweden’s food security.

## Materials and methods

### Case study: Sweden

**Fig. 1:**
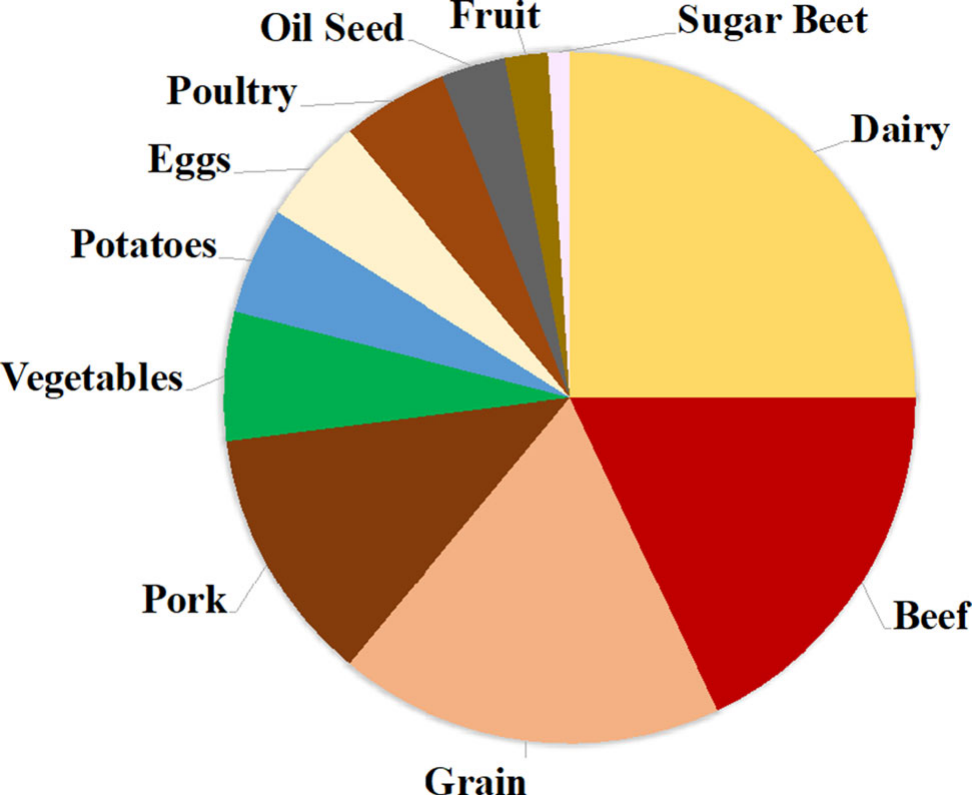
Swedish food production in 2016 (%) (adapted from Jordbruksverket 2017)

The Swedish population has increased by 2% annually since 1970, to the current 10 million people, 87% of whom live in cities (UN 2019). In the early 2000s, structural changes in Sweden’s agricultural sector resulted in a sharp decline in the number of farms, but an increase in farm size (Jordbruksverket 2017). These farms specialised, dedicating production to livestock (30%), dairy (25%), grains (18%) and vegetables (6%) (Fig. 1). In 2018, 2.7 million of the 3 million ha of arable land available in Sweden were cultivated, so the scope for expansion is limited (Jordbruksverket 2017; OECD 2020).

Increasing population, decreasing domestic production and changes in consumption behaviour (e.g. increasing demand for coffee, spices and more exotic products such as avocados) have resulted in Sweden becoming a net food importer (EC 2019; FAO 2020). In 2018, Swedish food imports were 6.6 billion USD and food exports represented 3.9 billion USD (WITS 2020). Within the EU, Sweden was a small food exporter in 2018 (e.g. Germany, the main EU exporter, distributed 46.9 billion USD) and the 10th largest EU importer of food, with ~ 9% of its total imports coming from partners outside Europe/Central Asia. The top three EU importers, the Netherlands (35%), Spain (29%) and Poland (24%), act as the main gatekeepers for imports from abroad into the EU (WITS 2020).

Although climate change is predicted to impact the agriculture system positively over the next few decades, this does not mean that food security in Sweden is safe. Migration to Sweden will accelerate the population increase and the population is predicted to reach 11.5 million by 2050, with 93% living in cities (UN 2019). Rapid expansion will add additional stress to the food system and, even if the predicted positive climate impacts occur, Sweden cannot grow all the products required (e.g. coffee, fruits) (Knight et al. 2007; Lundberg-Hállen and Öhrivik 2015).

### Assessment of Sweden’s trade partners

In food security assessments, we combined climate change, food and trade data to generate a Climate Vulnerability Index (CVI) for each food sub-category showing Sweden’s vulnerability to indirect climate impacts.

#### Statistics Sweden data

To provide context for Sweden’s current position as a net food importer, both globally and within the EU, data on 2000–2018 trade import shares for each of Sweden’s regional trade partners and each food import category were retrieved from Statistics Sweden (2019).

#### World integrated trade system database

The latest food import data (2018) were retrieved from the World Integrated Trade (WITS) database (2020), an online open-access data source reporting global and national trade values and quantities of different products for the period 1992–2018.

The WITS database provides information on food and non-food items, which can be customised based on country, indicator and product. For this study, searches were performed by product, and the results were customised to find gross imports into Sweden in 2018 from all countries.

Ten food categories and 28 food sub-categories were selected (Table 1). The WITS product categorisation guided the categories selected, but not all available data were included as only the natural forms of the products (before any preparations) were considered to be of interest (Appendix S1). For example, for the sub-category ‘Coffee’, only data for ‘Coffee: not roasted or decaffeinated’ were analysed. This provided a clearer understanding of Sweden’s relationship with its trade partners.

**Table 1:** Swedish food import categories and sub-categories included in the study

Categories		Sub-categories		
		Top imported food products		
		1	2	3
1	Seafood	Salmon	Cod	Shrimp
2	Meat and poultry	Beef	Poultry	Lamb
3	Coffee, Cacao and Tea	Coffee	Black tea	Cacao
4	Fruit	Bananas	Apples	Grapes
5	Vegetables	Tomatoes	Capsicum	Cucumber
6	Animal Products	Milk and cream	Eggs	–
7	Grains	Rice	Wheat	Maize
8	Nuts	Cashews	Coconuts	Pistachios
9	Spices	Black pepper	Paprika	Saffron
10	Sugar	Sugar cane	Sugar beet	–

Sub-categories 1, 2 and 3 represent the items with the first, second and third highest import value (kUSD)

To limit the computational effort, three sub-categories in each category representing the highest import values (kUSD) were selected. The ‘Animal Products’ and ‘Sugar’ categories only included two sub-categories, as these were the only two products available (Table 1). Monetary value was selected, since the larger the percentage of gross domestic product (GDP) invested in climate-vulnerable countries, the greater the vulnerability to indirect climate impacts (Benzie et al. 2019).

#### FAOSTAT database

The import values per trade partner for the 28 sub-categories were then used to calculate the trade partner import contribution (%) in 2018. To confirm that the trade partners identified for the sub-categories were producers of the food item, crop and livestock production source data in the FAOSTAT database were scrutinised. This revealed that certain trade partners were re-exporting partners, rather than actual producers of the food item (FAOSTAT 2018). As the aim of this study was to understand the indirect climate impacts from importing these items, knowing the source was crucial. To address this, Sweden’s trade partners were explored using import data available in the WITS database, following the methodology described in “World integrated trade system database” section.

Although the focus was on the top 10 partners, the top 20 partners needed to be included in order to ensure that significant import contributions, which could influence the final list of partners, were considered. Beyond the top 20 partners, import contributions became minimal (< 0.5) and did not affect the final list of source partners for these sub-categories.

### Calculating climate vulnerability

Climate vulnerability is the degree to which a system is susceptible to the effects of climate change (Parry et al. 2007). Vulnerability is a function of three dimensions: exposure, sensitivity and adaptive capacity. Weightings and assumptions on how the dimensions interact, and the use of past data or future predictions, can influence the results (Brook 2003; Downing et al. 2005). In this study, climate vulnerability is assessed using CVI, which is calculated as follows (Eq. 1) (Parry et al. 2007):

1 $${\text{CVI}} = \left( {E \times S} \right){-}A,$$

2 $$E = D_{{\text{o}}} + D_{{\text{r}}} + D_{i} ,$$

where *E* is exposure, *S* is sensitivity, *A* is adaptive capacity, *D*_o_ is dominance, *D*_r_ is direct trade and *D*_*i*_ is diversity, and all functions are given equal weighting.

#### Exposure

Exposure of a country’s food system to climate change depends on its reliance on climate-vulnerable countries (Benzie et al. 2019). The need for the three functions of exposure (Eq. 2) became apparent during the initial analysis of the WITS data.

Dominance (*D*_o_) refers to the number of source trade partners accounting for 75% of total imports for each food category. At least 75% of total imports for all sub-categories were accounted for by the top 10 partners. A score from 1 to 10 was attributed to each food sub-category, with 1 referring to one partner accounting for 75% of total imports and 10 referring to at least 10 partners. Direct trade (*D*_*i*_) describes the relationship between Sweden and its actual import partners. Each food sub-category was scored (0–10), with 1 referring to one partner being the source of the product and directly exporting to Sweden and 10 referring to 10 partners. Diversity (*D*_r_), based on the five climate zones proposed by Köppen (1936) (tropical, dry, temperate, continental, polar), reflects the capacity for a food sub-category to be produced in different regions and is based on the identified source partners (Beck et al. 2005). A sixth category was added to the five climate regions to account for food sub-categories produced in aquatic environments (both natural and aquaculture). Each food sub-category was scored (1–6), with 1 referring to production in one climate zone and 6 referring to production in all six climate zones.

The three Do, Di and Dr scores for each food sub-category were combined to give an exposure value ranging from 2 to 26, with 2 being the lowest possible score as diversity and dominance each required a minimum score of 1 for each exposure dimension. To comply with the conventional understanding of low and high exposure, the exposure values needed to be inverted so that a low value equated to low exposure of the food system to climate change. To produce comparable values, the inverted exposure scores were normalised by converting them to percentages.

#### Sensitivity

Sensitivity (S) is the degree to which a system is affected by exposure. The Climate Risk Index (CRI) (Eckstein et al. 2020) quantifies impacts of extreme weather events (related to precipitation and temperature) in terms of fatalities and economic losses arising from those events and provides a value for sensitivity. In this study, CRI scores recorded from 1999 to 2018 in 181 countries, extracted from Munich Re’s NatCatSERVICE on losses caused by natural disasters, such as floods and droughts (Eckstein et al. 2020), were used.

Scores were calculated from a country’s weighted average ranking in four categories: number of deaths, number of deaths per 100 000 inhabitants, sum of losses (USD) in purchasing power parity and losses per unit GDP (Eq. 3) (Eckstein et al. 2020):

3 $${\text{CRI}} = \left( {F.1/6} \right) + \left( {I.1/3} \right) + \left( {A.1/6} \right) + \left( {L.1/3} \right),$$

where *F* is fatalities toll ranking, *I* is fatalities per 100 000 inhabitants, *A* is absolute losses (million USD PPP) and *L* is losses per unit GDP.

The CRI includes both absolute and relative impacts to calculate an average ranking of countries in four indicator categories, with a focus on the relative indicators (*I*, *L*). The weighting gives preference to *I* and *L* since their values, and the final score, undergo change not only due to absolute impacts of extreme weather events (direct impacts) but also to population and economic changes (indirect impacts) (Eckstein et al. 2020). The final CRI values used here to assess the sensitivity of Sweden’s trade partners were based on the relative score awarded to each country. An average CRI score for the source trade partners was calculated and used as the sensitivity value for each sub-category. As with exposure, the values were inverted and normalised.

#### Adaptive capacity

Adaptive capacity (*A*) is the ability of a system to adjust to climate change due to access to financial, technical, educational and community resources (Brooks 2003; Benzie et al. 2019). The Fragile State Index (FSI), developed by Fund for Peace (2020), provides an assessment of the vulnerability of 178 countries through 12 indicators addressing five main themes (Table 2).

**Table 2:** Description of Fragile State Index (FSI) score indicators ( adapted from Fund for Peace 2020)

Themes	Indicators	Description
Cohesion	Security Apparatus	Security threats faced by a state. Includes the control over force, security and citizenry relationship, and force and arms use
	Factionalised Elites	Fragmentation of state institutions. Includes representative leadership, resource distribution and equality
	Group Grievances	Political and societal differences in society. Includes post-conflict responses, equality levels, divisions and communal violence
Economics	Decline and Poverty	Economic decline within a country. Includes public finances, economic climate and diversification
	Uneven Development	Inequality within the economy. Includes economic equality, opportunity and socio-economic dynamics
	Human Flight and Brain Drain	Economic impact of human displacement. Includes retention of intellectual and technical capital, economics and diaspora
Political	State Legitimacy	Government relationship with citizens. Includes confidence in government, opposition, transparency, openness of political process
	Public Services	Basic state services. Includes provision of public services, health, education and infrastructure
	Human Rights and Rule of Law	State and citizens relationships. Includes civil and political rights, justice and violation of rights
Social	Demographic Pressures	Population pressures on State. Includes population, health, food, environment and other resources
	Refugees	Pressures caused by forced displacement. Includes responses to displaced groups
Cross-cutting Topics	External Intervention	External influences on the state. Includes political and economic intervention

To assess adaptive capacity, the Conflict Assessment System Tool (CAST) was employed. This tool places emphasis on the use of both qualitative and quantitative data. In initial content analysis, Boolean search phases, based on the 12 indicators (Table 2), were applied to global media data (news reports and academic articles) collected through a commercial content aggregator. Based on the assessed saliency, provisional scores were awarded to each country. Quantitative data on key aspects of the 12 indicators were then gathered from multinational agencies, such as the World Bank and World Health Organisation. Based on the extent to which the quantitative assessment scores matched the content analysis, the scores were either confirmed or re-evaluated. A qualitative review provided assessments based on prior results. The results were then triangulated and subjected to critical review, to address any gaps or biases.

An FSI score was awarded to each of the 178 countries and the countries were then ranked, with a higher score identifying a less fragile country. The average FSI score for the source trade partners was used to produce an adaptive capacity score for each food sub-category. The adaptive capacity values were not inverted, as a higher value represented high adaptive capacity, but the values were normalised.

## Results

**Fig. 2:**
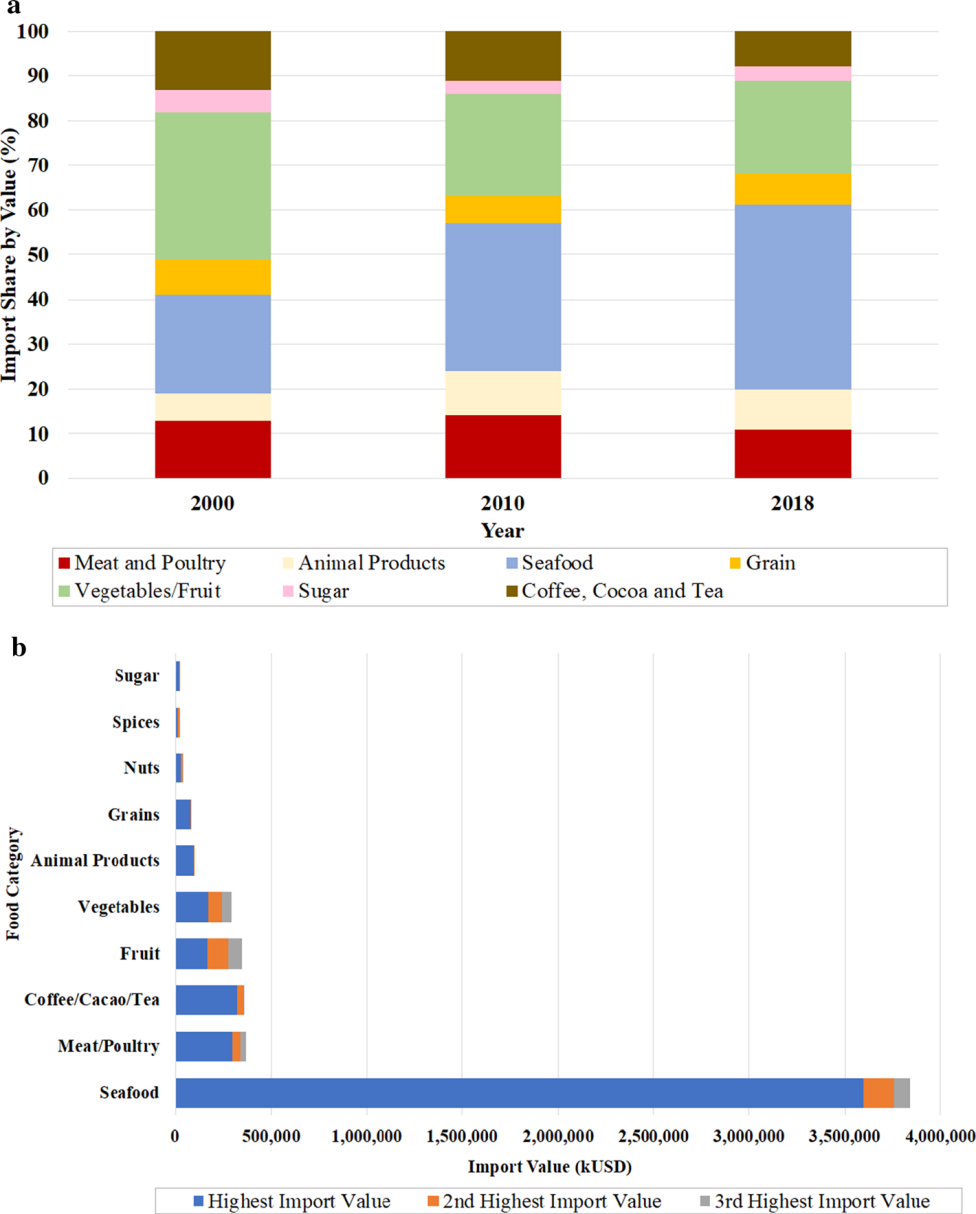
**a** Contribution of the 10 top food import categories in Sweden in 2000, 2010 and 2018 to total Swedish food imports and **b** overall import value of the top Swedish food imports for each food category and their sub-categories in 2018 (based on Statistics Sweden 2019)

Figure 2a provides an overview of the main food product imports into Sweden between 2000 and 2018. Seafood products and vegetables/fruit were the largest categories of imported products. Seafood imports almost doubled, from 22% in 2000 to 41% in 2018, whilst vegetables/fruit imports decreased from 32% in 2000 to 21% of total food imports in 2018. Meat and poultry imports decreased slightly (~ 2%), whilst imports of animal products (milk and cream, and eggs) increased by 3%. Grain imports accounted for 7% of total food imports in 2018. Coffee, cocoa, tea and sugar had low import shares and displayed steady decreases from 2010 onwards.

In terms of the import values (kUSD) of the food sub-categories included in this study, seafood products, in particular, salmon, accounted for the highest import value in 2018 (Fig. 2b).

**Fig. 3:**
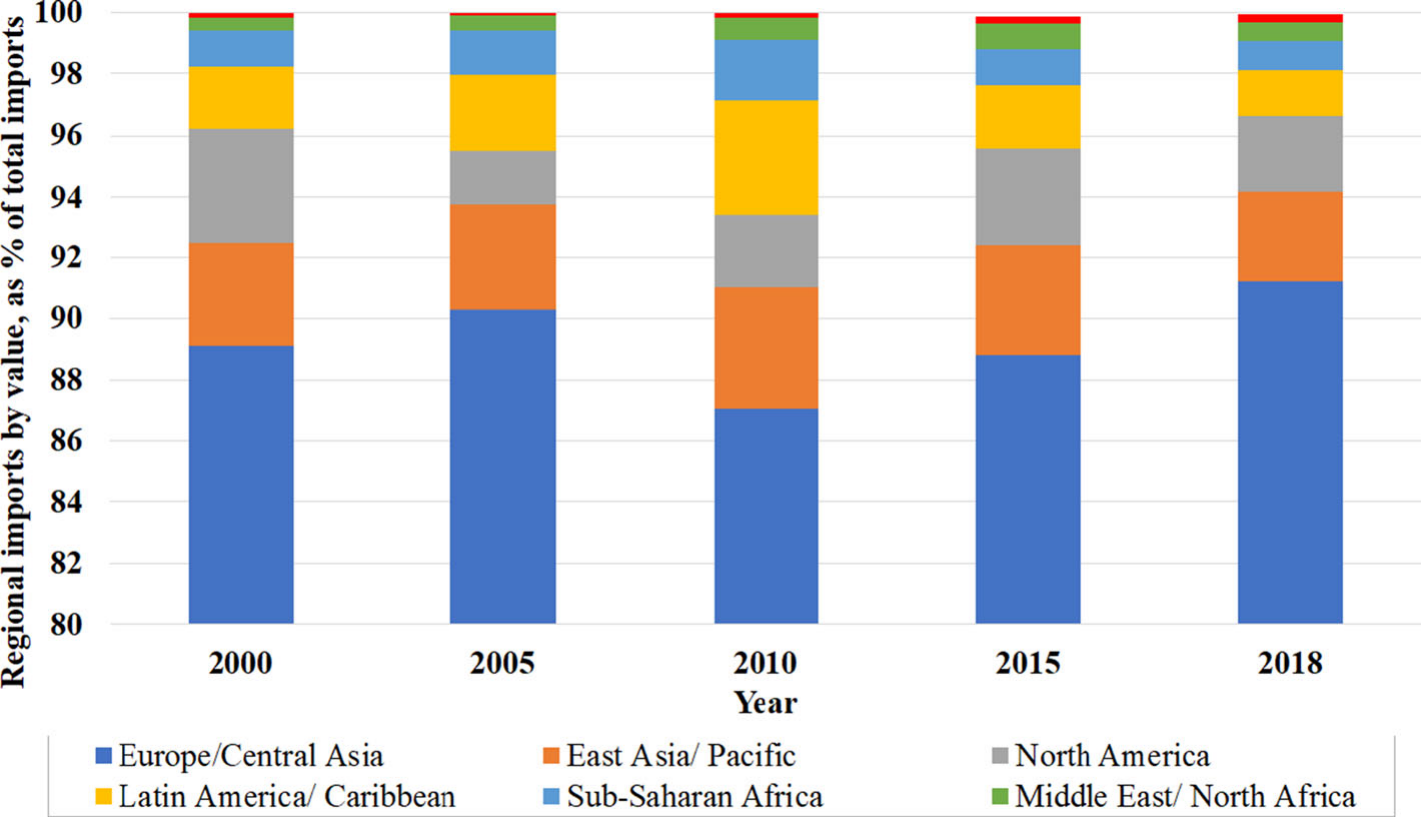
Regional food imports to Sweden in 2018 by value, as a percentage of total imports (based on Statistics Sweden 2019)

Europe/Central Asia has been Sweden’s main partner in the past two decades, providing 87–91% of annual food imports. North America was second largest in 2000 (4%), but East Asia/Pacific was the second largest in 2018 (3%) (Fig. 3).

**Fig. 4:**
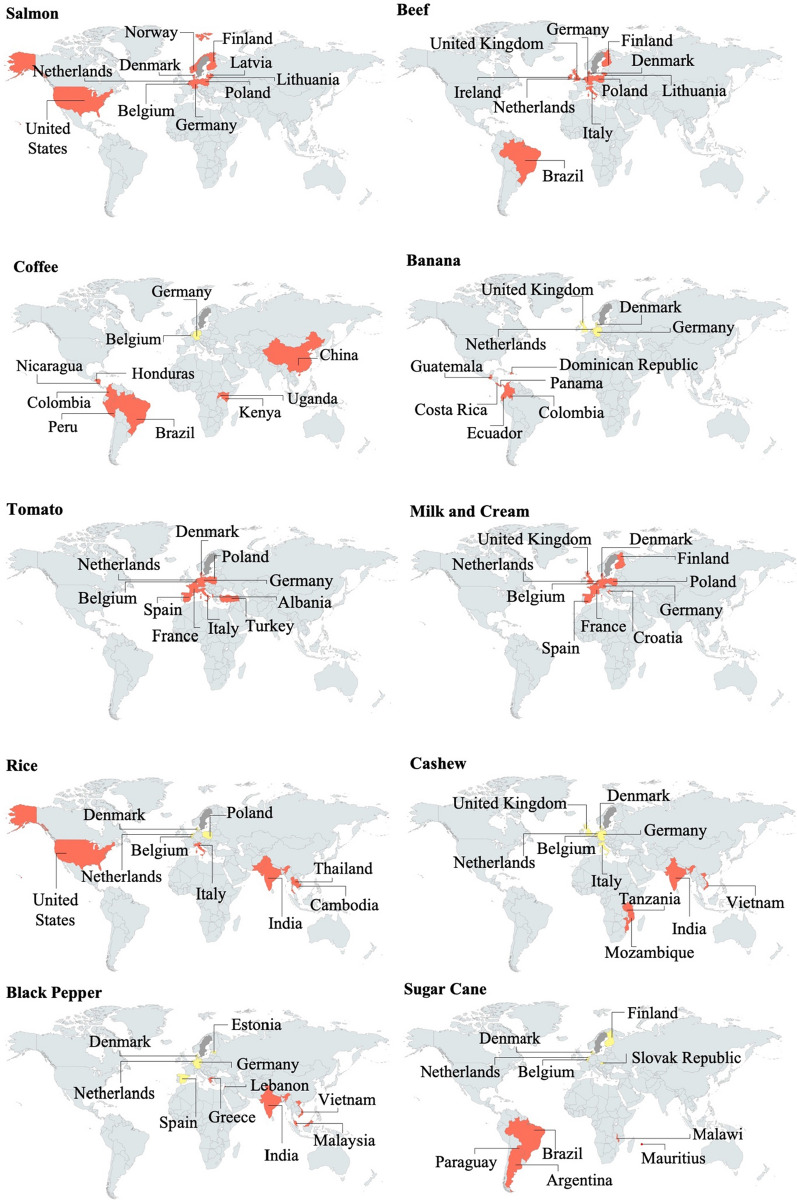
Sweden’s main trade partners for the sub-category of each of the 10 food categories with the highest import value in 2018. Partners which both produce and export are shown in red, re-exporting partners are in yellow

The global distribution of Sweden’s top trade partners for each of the highest import value food sub-categories is shown in Fig. 4. These sub-categories illustrate the diversity and dominance of Sweden’s partners and their trade pathways. Appendix S2 details the second and third highest imports of the 18 food sub-categories.

Table 3 shows the overall individual climate vulnerability scores for each of the 28 food sub-categories and the overall score for the 10 food categories. The CVI scores are based on level of exposure, sensitivity, adaptive capacity and vulnerability to climate change. Nuts were found to have the highest CVI score, whilst animal products had the lowest.

**Table 3:**
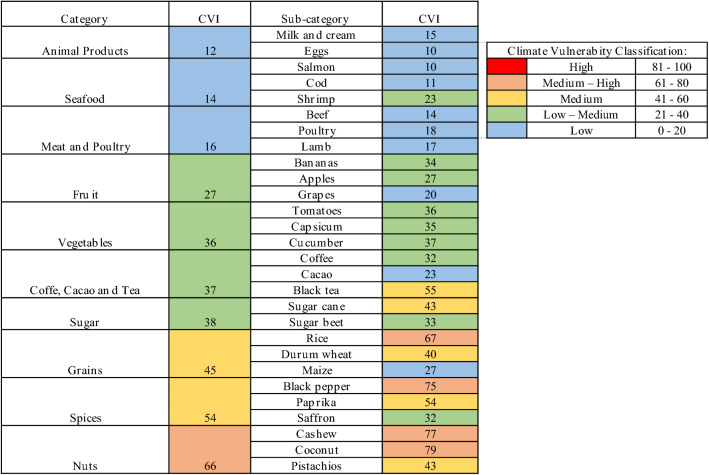
Climate Vulnerability Index (CVI) values for the 10 categories and 28 sub-categories of food imports to Sweden in 2018

## Discussion

### A net food importer: Sweden and its main trade partners

The 28 food sub-categories imported in 2018 were sourced from 74 countries (Fig. 4). Thirty-four percent were European, 22% African, 22% South American and Caribbean, 12% Asian, 8% Middle Eastern, 1% North American and 1% from the Oceania region (Fig. 3). Sweden’s position as a net importer, reliant on countries with a broad range of potential climate risk due to their varying geographical, societal, economic and political situations, raises concerns regarding its food system vulnerabilities.

### Climate vulnerability—implications for food availability

#### Food categories with low vulnerability

Animal products had the lowest vulnerability to climate change (CVI = 12), closely followed by seafood (CVI = 14), and meat and poultry (CVI = 16) (Table 3). These low CVI scores arose because the identified trade partners had low exposure and sensitivity scores, and high adaptivity potential.

As animal proteins play a major role in Swedish diets, the CVI scores are reassuring. However, the intensification of livestock production to meet growing demand, paired with rising temperatures, will have negative effects on livestock production and on animal welfare (Gomez-Zaglavia et al. 2020). In temperate climates, covering part of Sweden, warming is expected to lengthen the forage growing season but decrease forage quality, creating further challenges (Hoegh-Guldberg et al. 2018).

The majority of Sweden’s seafood is sourced from mid- to high-latitude countries. Fisheries located in these regions are predicted to experience increased productivity, due to a shift of species towards higher latitudes (Hoegh-Guldberg et al. 2018). For Sweden’s main trading partners, warming, increased light levels and mixing from retreating sea ice could result in increases in fish productivity in the North Atlantic (Hoegh-Guldberg et al. 2018). However, there is a risk of disease and invasive species (Hoegh-Guldberg et al. 2018).

To address these concerns, more resilient fish breeds, cooling technologies and increased measures to protect fish stocks in trade partners’ areas could be explored (Gomez-Zaglavia et al. 2020). If the climate permits, a shift in diet towards local and seasonal food is another possibility (Gomez-Zaglavia et al. 2020).

#### Food categories with low to medium vulnerability

Fruit (CVI = 27), vegetables (CVI = 36), coffee, cocoa and tea (CVI = 37) and sugar (CVI = 38) showed low–medium vulnerability to climate change. Within these categories, the sub-categories varied between low and medium vulnerability.

For fruit and vegetables, the majority of the sub-categories had low–medium vulnerability to climate change. Bananas had the highest exposure value and the lowest sensitivity value, but also the lowest adaptive capacity, due to reliance on trade with countries such as Ecuador and the Dominican Republic (Fig. 4). The impact of climate change on Central and South America, over the next few decades, will vary between regions. In the sub-tropical and tropical regions where bananas are grown, increases in temperature extremes have been predicted for Central America and most tropical and subtropical regions of South America (Hoegh-Guldberg et al. 2018). Climate predictions may be more favourable for banana production in Ecuador, but production in many African countries and Central American countries is at risk (Varma and Bebber 2019). A decrease in global supply would put added pressure on those countries able to produce and perhaps increase Sweden’s competition for Ecuadorian bananas (Varma and Bebber 2019).

Local crops cultivated in specific climate conditions are particularly affected by climate change (Hoegh-Guldberg et al. 2018). Tea showed higher vulnerability due to its high sensitivity score, low adaptive capacity and low climate diversity scores. Coffee and cocoa were classified as low–medium vulnerability.

Sweden imports both Arabica and Robusta coffee, but the market for the latter is limited (CBI 2020). If Brazil, Sweden’s main Arabica bean trade partner (Fig. 4) and the world’s largest coffee producer, experiences decreasing crop yields or a reduction in quality due to climate-related challenges, Sweden’s options for obtaining coffee would decrease. Climate scenarios indicate that countries such as Brazil may become too dry and hot to permit productive coffee production, particularly given the limited potential for irrigation in countries already suffering from water scarcity (EC 2020). Adaptation strategies, such as relocating coffee farms, need to be considered (Hoegh-Guldberg et al. 2018).

#### Food categories with medium–high and high vulnerability

Grains (CVI = 45) and spices (CVI = 54) had medium–high vulnerability, whilst nuts (CVI = 66) had high vulnerability to climate change.

Staple crops have greater potential for vulnerability driven by indirect climate impacts. In 2017, Sweden produced 3 million tonnes of winter wheat (Jordbruksverket 2018). In the following year, the 2018 European heatwave hit and Sweden was only able to produce 1.4 million tonnes, leaving it unable to meet domestic demand (Jordbruksverket 2019). Inability to turn to other EU countries for supplies, as their yields were also affected, left Sweden exposed. Diversification of key trade partners, particularly to include countries with different current and predicted future climates, should therefore be considered. However, global temperature and precipitation trends are already having negative impacts on wheat and maize crops, meaning that future diversification may not be easy (Hoegh-Guldberg et al. 2018). Winter wheat production in Sweden recovered rapidly after the 2018 heatwave and in 2019, 3.3 million tonnes were produced (Jordbruksverket 2020). However, if extreme weather events increase in frequency and intensity, the timeframe to ‘bounce back’ will be reduced.

Rice had a very high vulnerability score and, in addition to its low diversity and dominance scores, was also affected by a low direct trade score. If a rice shortage occurs, reliance on one partner and on re-exporting partners would leave Sweden highly vulnerable. As demonstrated during the COVID global pandemic, governments can restrict food exports, disrupting food systems (EC 2020). Trading restrictions imposed during 2020 affected 1% of Sweden’s imported calories, but e.g. 79% of Tajikistan’s (Coghlan et al. 2014; IFPRI 2020).

Black pepper, cashews and coconuts had the highest CVI scores. This low adaptive capacity demonstrated the fragility of the relevant trade partners, but also the inevitable vulnerability because of the limited regions in which these crops can be grown (Hoegh-Guldberg et al. 2018). Although not staples, items such as these are significant in the food culture of Sweden and many other developed countries, so their vulnerability should not be overlooked (Lundberg-Hállen and Öhrvik 2015).

This study demonstrated the complexity of assessing food security vulnerability due to climate change. It showed that different scores can be obtained through a range of different pathways, providing a platform from which to consider Sweden’s management options (Ginbo et al. 2020). Sweden’s pedoclimatic properties and its increasing population require the government to look beyond its borders and offer climate mitigation/adaptation support to the key partners.

### Data limitations

The WITS database lacks details on the exact percentage of a food item produced in a country, which made it difficult to calculate the exact contribution of each trade partner. A similar limitation arose with the CEPII database, demonstrating that the problem lies in how data are recorded by these large databases.

This study identified a need for more comprehensive data to support further analysis. Information on the impacts of climate change on different crops is needed, so that food security can be investigated more accurately. To further address the complex issues raised in this paper, the links between food security, trade and climate change should be examined.

### Policy recommendations and future research

This study revealed some of the hidden links between climate change and the global food trading system. Based on CVI scores, an early warning system about insecurities in the food system could be developed (Gomez-Zavaglia et al. 2020). The CVI scores for all imported food items should be regularly updated and IPCC climate predictions should be included when monitoring vulnerability. The CVI methodology could be improved for particular sub-categories and Sweden’s entire network of trade partners could be included in future assessments of current vulnerability and comparisons of CVI. More detailed knowledge of food system vulnerabilities to climate change is required to develop policies and measures which can help achieve the SDG2 targets.

To determine how climate vulnerability impacts trade patterns, the results from this study could be integrated into the ‘Gravity’ model, together with data from the CEPII database. In its basic form, the Gravity model holds that a mass of goods (or other factors of production) supplied at an origin is attracted to a mass of demand for goods at a destination, but that the potential flow is reduced by the distance between origin and destination (Anderson 2011; Backhaus and Martínez-Zarzoso 2015). The basic model could be expanded to include different variables, to test whether they are relevant in explaining trade patterns, extending the work in this study.

## Conclusion

Food security in Sweden and some other developed countries relies mostly on imports. Amongst the main imported food categories, grain, nuts and spices are the most vulnerable to climate change, whilst animal products are the least vulnerable. Quantitative analysis of emerging climate impact provides a new perspective on how indirect climate impacts can affect a country’s food security. Globalisation has played a role in assuring food security in many countries, but to achieve the Agenda 2030 SDG2 and effectively manage the risks, climate change and food trade pathways must be evaluated together and the results must be considered in policy formulation and decision-making.

## Supplementary Information

Below is the link to the electronic supplementary material.

Supplementary file1 (PDF 575 kb)
