# Supplementary material for: Links between food trade, climate change and food security in developed countries: A case study of Sweden
Source: Ambio. 2021 Sep 24;51(4):943–54. doi: 10.1007/s13280-021-01623-w (PMC8847661; doi:10.1007/s13280-021-01623-w)
Supplement: Supplementary file 1 — Supplementary file1 (PDF 575 kb) [file 13280_2021_1623_MOESM1_ESM.pdf]

Ambio

Electronic Supplementary Material
This supplementary material has not been peer reviewed.

Title: **Climate Vulnerability through Food Trade in Developed Countries: A Case Study of Sweden**

Authors: Blaze Horn, Carla Ferreira, Zahra Kalantari

Appendix S1. *Food categories from the WITS database included in each sub-category considered in the current study.*

Category	Sub-Category	WITS Database code and category
Seafood	Salmon	030212 -- Fish; Pacific salmon (oncorhynchus spp.), Atlantic salmon (salmo salar), Danube salmon (hucho hucho), fresh or chilled (excluding fillets, livers, roes and other fish meat of heading no. 0304)
	Cod	030250 -- Fish; cod (gadus morhua, gadus ogac, gadus macrocephalus), fresh or chilled (excluding fillets, livers, roes and other fish meat of heading no. 0304)
	Shrimp	030623 -- Crustaceans; shrimps and prawns, not frozen, (whether in shell or not, whether or not cooked by steaming or by boiling in water)
Meat and Poultry	Beef	020110 -- Meat; of bovine animals, carcasses and half-carcasses, fresh or chilled
	Lamb	020410 -- Meat; of sheep, lamb carcasses and half-carcasses, fresh or chilled
	Poultry	020710 -- Meat and edible offal; poultry, not cut in pieces, fresh or chilled
Coffee, Cacao and Tea	Coffee	090111 -- Coffee; not roasted or decaffeinated
	Cacao	180100 -- Cocoa beans; whole or broken, raw or roasted
	Black Tea	090220 -- Tea, green; (not fermented), in immediate packings of a content exceeding 3kg
Fruit	Banana	080300 -- Fruit, edible; bananas, (including plantains), fresh or dried
	Apple	080810 -- Fruit, edible; apples, fresh

	Grape	080610 -- Fruit, edible; grapes, fresh
Vegetables	Tomato	070200 -- Vegetables; tomatoes, fresh or chilled
	Capsicum	070960 -- Vegetables; fruits of the genus capsicum or of the genus pimenta
	Cucumber	070700 -- Vegetables; cucumbers and gherkins, fresh or chilled
Animal Products	Milk and Cream	040110 -- Dairy produce; milk and cream, not concentrated, not containing added sugar or other sweetening matter, of a fat content not exceeding 1% (by weight)<br><br>040120 -- Dairy produce; milk and cream, not concentrated, not containing added sugar or other sweetening matter, of a fat content exceeding 1% but not exceeding 6% (by weight)<br><br>040130 -- Dairy produce; milk and cream, not concentrated, not containing added sugar or other sweetening matter, of a fat content exceeding 6% (by weight)
	Egg	040700 -- Eggs; birds' eggs, in the shell, fresh, preserved or cooked
Grains	Rice	100610 -- Cereals; rice in the husk (paddy or rough)<br>100620 -- Cereals; husked (brown) rice<br><br>100630 -- Cereals; rice, semi-milled or wholly milled, whether or not polished or glazed
	Wheat	100110 -- Cereals; durum wheat
	Maize	100510 -- Cereals; maize (corn), seed
Nuts		080130 -- Nuts, edible; cashew nuts, fresh or dried, whether or not shelled or peeled

	Cashews	
	Coconut	080110 -- Nuts, edible; coconuts, fresh or dried, whether or not shelled or peeled
	Pistachios	080250 -- Nuts, edible; pistachios, fresh or dried, whether or not shelled or peeled
Spices	Black Pepper	090411 -- Spices; pepper (of the genus piper), neither crushed nor ground
	Paprika	090420 -- Spices; fruits of the genus capsicum or pimenta, dried or crushed or ground
	Saffron	091020 -- Spices; saffron
Sugar	Sugar Cane	170111 -- Sugars; cane sugar, raw, in solid form, not containing added flavouring or colouring matter
	Sugar Beet	170112 -- Sugars; beet sugar, raw, in solid form, not containing added flavouring or colouring matter

Appendix S2. *Global trade Maps for the 18 sub-categories with the 2nd and 3rd highest import values in 2018. Sweden Trade Partners which both produce and export are shown in red, re-exporting partners are in yellow.*

Poultry

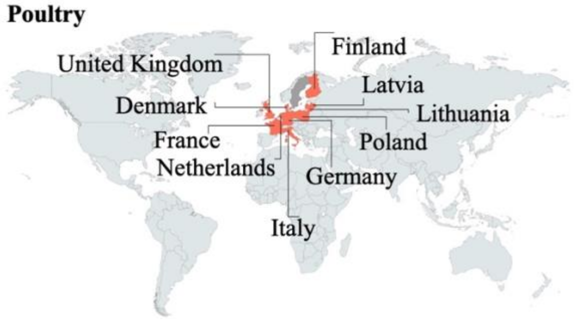

Lamb

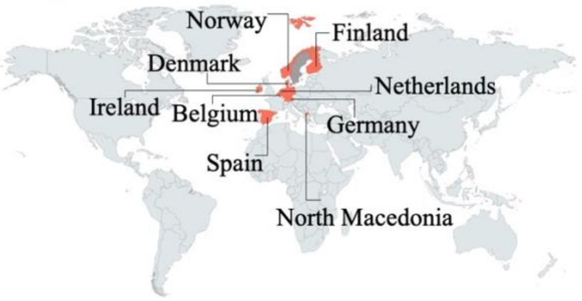

Cod

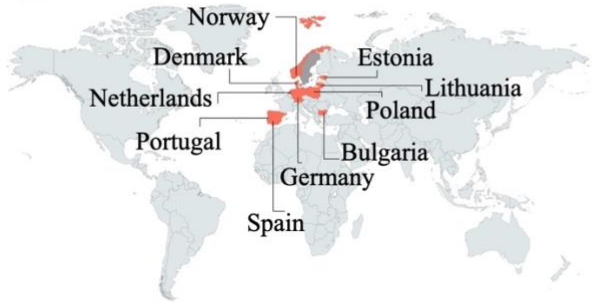

Shrimp

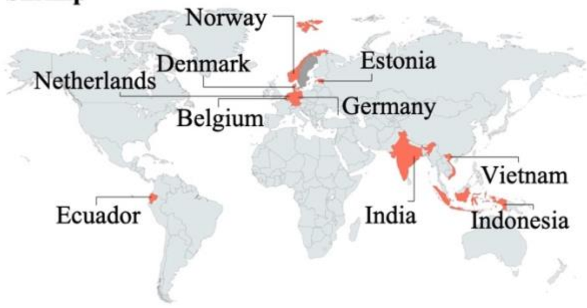

Cacao

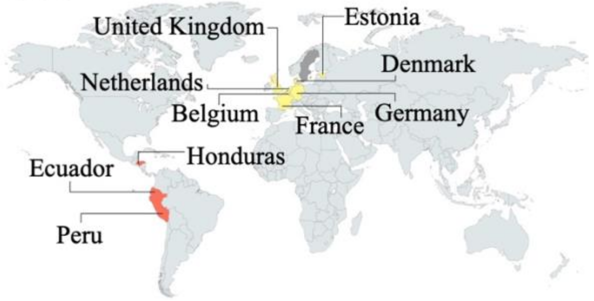

Black Tea

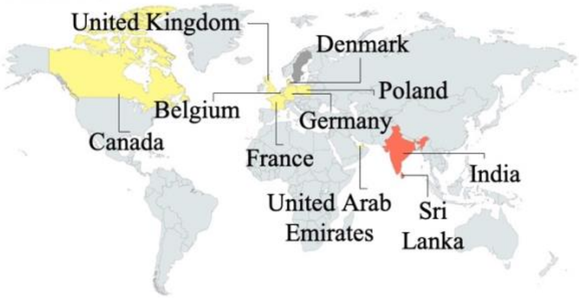

Egg

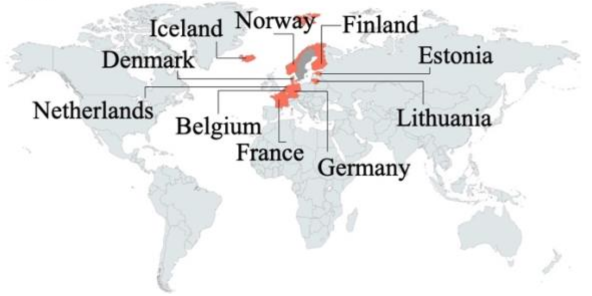

Sugar Beet

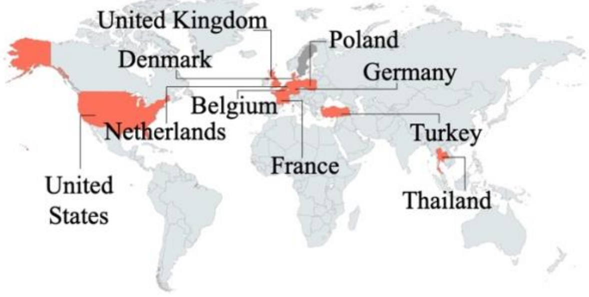

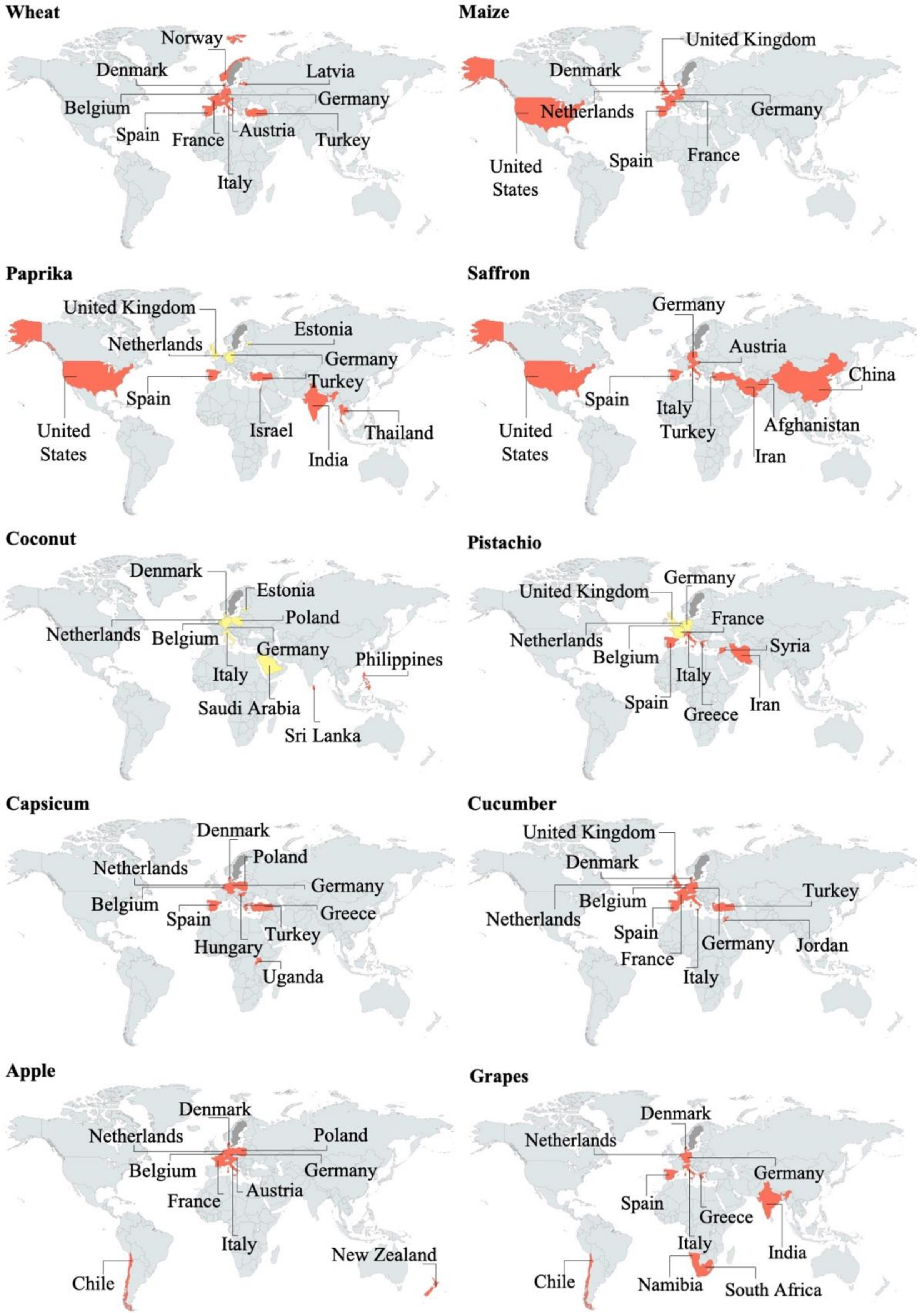
Wheat
Norway
Denmark
Latvia
Germany
Belgium
Spain
France
Austria
Turkey
Italy
Maize
United Kingdom
Denmark
Netherlands
Germany
United States
Spain
France
Paprika
United Kingdom
Estonia
Netherlands
Germany
Turkey
Spain
United States
Israel
Thailand
India
Saffron
Germany
Austria
China
Spain
Italy
Turkey
Afghanistan
Iran
United States
Coconut
Denmark
Estonia
Poland
Netherlands
Belgium
Germany
Philippines
Italy
Saudi Arabia
Sri Lanka
Pistachio
Germany
United Kingdom
France
Netherlands
Belgium
Syria
Italy
Spain
Iran
Greece
Capsicum
Denmark
Poland
Netherlands
Germany
Belgium
Spain
Greece
Turkey
Hungary
Uganda
Cucumber
United Kingdom
Denmark
Belgium
Turkey
Netherlands
Spain
Germany
Jordan
France
Italy
Apple
Denmark
Netherlands
Poland
Belgium
Germany
Austria
France
Italy
New Zealand
Chile
Grapes
Denmark
Netherlands
Germany
Spain
Greece
Italy
India
Chile
Namibia
South Africa

Appendix S3. *Results of the CVI and the associated three dimensions (exposure, sensitivity and adaptive capacity) scores.*

Category	CVI	Sub-category	CVI	CVI (Normalised)	Exposure						Sensitivity			Adaptive Capacity	
					Dominance	Direct Trade	Diversity	Total Exposure	Inverted	Normalised	CRI	Inverted	Normalised	FSI	Normalised
		Milk and Cream	13	15	2	10	4	16	10	38	113	68	38	174	98
Animal Products	12	Eggs	9	10	2	10	4	16	10	38	135	46	25	174	98
		Salmon	9	10	1	10	1	12	14	54	148	33	18	177	99
		Cod	10	11	1	10	1	12	14	54	145	36	20	176	99
Seafood	14	Shrimp	21	23	1	10	1	12	14	54	106	75	41	162	91
		Beef	13	14	5	10	4	19	7	27	86	95	52	156	88
		Poultry	17	18	3	10	4	17	9	35	89	92	51	166	93
Meat and Poultry	16	Lamb	15	17	2	10	4	16	10	38	104	77	43	169	95
		Bananas	31	34	3	6	1	10	16	62	88	93	51	99	56
		Apples	25	27	6	10	1	17	9	35	48	133	73	145	81
Fruit	27	Grapes	19	20	4	10	4	18	8	31	67	114	63	154	87
		Coffee	30	32	6	8	1	15	11	42	52	129	71	75	42
		Cacao	21	23	5	2	1	8	18	69	125	56	31	50	28
Coffe, Cacao and Tea	37	Black Tea	51	55	4	5	1	10	16	62	30	151	83	56	31
		Sugar Cane	40	43	2	5	1	8	18	69	75	106	59	100	56
Sugar	38	Sugar Beet	30	33	1	10	1	12	14	54	76	105	58	149	84
		Tomatoes	33	36	2	10	2	14	12	46	48	133	73	159	89
		Capcisum	32	35	2	10	2	14	12	46	53	128	71	155	87
Vegetables	36	Cucumber	34	37	2	10	2	14	12	46	45	136	75	145	81
		Rice	62	67	1	4	2	7	19	73	26	155	86	142	80
		Durum Wheat	37	40	2	10	2	14	12	46	34	147	81	157	88
Grains	45	Maize	25	27	2	10	1	13	13	50	87	94	52	171	96
		Black Pepper	69	75	1	3	1	5	21	81	25	156	86	78	44
		Paprika	50	54	4	5	1	10	16	62	32	149	82	115	65
Spices	54	Saffron	29	32	2	10	3	15	11	42	53	128	71	112	63
		Cashew	70	77	1	4	1	6	20	77	14	167	92	98	55
		Coconut	72	79	2	2	1	5	21	81	18	163	90	54	30
Nuts	66	Pistachios	40	43	3	5	1	9	17	65	69	112	62	84	47

Climate Vulnerability Classification:		
	**High**	81 - 100
	**Medium - High**	61 - 80
	**Medium**	41 - 60
	**Low - Medium**	21 - 40
	**Low**	0 - 20

References

Eckstein, D., Künzel, V., Schäfer, L., and Winges, M. 2020. Global Climate Risk Index 2020. Germanwatch.

Fund for Peace. 2020. Fragile State Index. Retrieved 1 September 2020, from https://fragilestatesindex.org/data/

World Integrated Trade Solution (WITS). 2020. Trade Database. Retrieved 1 September 2020, from https://wits.worldbank.org/
